# (-)-T-Cadinol—a Sesquiterpene Isolated From *Casearia sylvestris* (Salicaceae)—Displayed *In Vitro* Activity and Causes Hyperpolarization of the Membrane Potential of *Trypanosoma cruzi*

**DOI:** 10.3389/fphar.2021.734127

**Published:** 2021-11-03

**Authors:** Augusto L. dos Santos, Maiara Amaral, Flavia Rie Hasegawa, João Henrique G. Lago, Andre G. Tempone, Patricia Sartorelli

**Affiliations:** ^1^ Instituto de Ciências Ambientais, Químicas e Farmacêuticas, Universidade Federal de São Paulo, Diadema, Brazil; ^2^ Centro de Parasitologia e Micologia, Instituto Adolfo Lutz, Santo André, Brazil; ^3^ Faculdade de Medicina, Universidade de São Paulo, São Paulo, Brazil; ^4^ Centro de Ciências Naturais e Humanas, Universidade Federal do ABC, Santo André, Brazil

**Keywords:** *Trypanosoma cruzi*, *Casearia sylvestris*, Salicaceae, T-cadinol, Chagas disease

## Abstract

Chagas disease is caused by the protozoan parasite *Trypanosoma cruzi* and affects 6–8 million people worldwide, mainly from developing countries. The treatment is limited to two approved nitro-derivatives, nifurtimox and benznidazole, with several side effects and reduced efficacy. *Casearia sylvestris* has been used in folk medicine as an antiseptic and cicatrizing in skin diseases. In the present work, the hexane phase from the MeOH extract from the leaves of *Casearia sylvestris* afforded a fraction composed by the sesquiterpene T-cadinol, which was chemically characterized by NMR and HRMS. The activity of T-cadinol was evaluated against *T. cruzi*, and IC_50_ values of 18 (trypomastigotes) and 15 (amastigotes) µM were established. The relation between the mammalian toxicity and the antiparasitic activity resulted in a selectivity index >12. Based on this promising activity, the mechanism of action was investigated by different approaches using fluorescent-based techniques such as plasma membrane permeability, plasma membrane electric potential, mitochondrial membrane electric potential, reactive oxygen species, and the intracellular calcium (Ca^2+^) levels. The obtained results demonstrated that T-cadinol affected neither the parasite plasma membrane nor the electric potential of the membrane. Nevertheless, this compound induced a mitochondrial impairment, resulting in a hyperpolarization of the membrane potential, with decreased levels of reactive oxygen species. No alterations in Ca^2+^ levels were observed, suggesting that T-cadinol may affect the single mitochondria of the parasite. This is the first report about the occurrence of T-cadinol in *C. sylvestris*, and our data suggest this sesquiterpene as an interesting hit compound for future optimizations in drug discovery studies for Chagas disease.

## Background

Neglected tropical diseases (NTDs) are a group of parasitic diseases that affect more than one billion people worldwide, corresponding to 12% of the global health burden, and generating a massive social, as well as, economic impact (Who, 2017). Among the NTDs, Chagas disease (CD) is considered endemic, with approximately 14 thousand deaths per year (De Rycker et al., 2018). The etiologic agent of CD is the kinetoplastid *Trypanosoma cruzi*, which could be detected as extracellular (trypomastigote) or intracellular (amastigote) forms. In humans, the intracellular amastigotes can persist for decades, leading to a severe cardiomegaly and damage to the digestive tract (megacolon) (Kratz, 2019). Moreover, current therapies for CD were introduced in the 1970s and comprise benznidazole and nifurtimox (Ribeiro et al., 2020), with several side effects and reduced efficacy, especially in the chronic phase of this disease. Therefore, there is an urgent need of new alternative treatments for CD, including compounds, which could be obtained from natural sources such as plants, animals, and microorganisms (Newman and Cragg, 2020).

*Casearia*, belonging to Salicaceae, is popularly known in Brazil as “guaçatonga,” a term originated from the Tupi–Guarani language, indicating an ancient use of this species in Brazilian indigenous communities (Ferreira et al., 2011; Grandi, 2014; Ameni et al., 2015). In this genus, there are more than 50 different species, but just few of these were evaluated from a pharmacological or chemical point of view (Xia et al., 2015; Marquete Mederios, 2020). The ethnopharmacological uses of *C. sylvestris* include antiseptic and cicatrizing in skin diseases, anti-inflammatory, anti-ophidic, anti-pyretic, and anti-ulcer effects; this potential is frequently associated to the presence of oxidized clerodane diterpenes known as casearins and caseavestrins (Itokawa et al., 1990; Ferreira et al., 2011; Ferreira et al., 2014; Xia et al., 2015). The antiparasitic potential of diterpenoids from *C. sylvestris* was previously reported, including the *in vitro* action of the leaves extract (Mesquita et al., 2005; Antinarelli et al., 2015) and casearins A, B, G, and J against *Leishmania* spp*.* and *T. cruzi* (Bou et al., 2014). Casearin X, one of the main compounds found in *C. sylvestris*, also displayed *in vitro* cytotoxic activity against several cell lines including antitumor potential in preclinical assays (Xia et al., 2015; Ferreira et al., 2016).

In the present work, the fractionation of the hexane phase obtained from the MeOH extract from the leaves of *C. sylvestris* afforded the sesquiterpene T-cadinol, which was chemically characterized by nuclear magnetic resonance (NMR), mass spectrometry (MS) analysis, and polarimetric data. The antitrypanosomal activity of this compound was evaluated in the extracellular trypomastigotes and intracellular amastigotes of *Trypanosoma cruzi*. A mechanism of action study was also performed, using spectrofluorimetric and flow cytometry assays to evaluate the plasma membrane permeability, the electric potential of the plasma membrane, the mitochondrial membrane potential, reactive oxygen species (ROS) levels, and the intracellular calcium levels. In trypanosomatids, these organelles have been extensively studied in drug discovery as essential sites for targeting new drug candidates.

## Materials and Methods

### General Experimental Section

^1^H and ^13^C NMR spectra were recorded at 300 and 75 MHz, respectively, on a Bruker Ultrashield 300 Advance III spectrometer (Bruker–Biospin, Germany). DMSO (Aldrich) was used as the solvent and as the internal standard. ESI–HRMS were obtained in a Bruker Daltonics microTOF-QII (ESI–QTOF positive mode). Silica gel (Merck, 230–400 mesh) and Sephadex LH-20 were used for the chromatographic column (CC) separation, while silica gel (SiO_2_) 60 PF_254_ (Merck) was used for analytical thin layer chromatography.

### Plant Material

The leaves of *C. sylvestris* were collected from a single tree in the Atlantic Forest district of São Paulo City, SP, Brazil (coordinates 23 53′08.86″S, 46 40′10.45″O), in October 2012. Botanical identification was made by Dr. Roseli Buzanelli Torres, and the leaves were deposited into the IAC Herbarium with the voucher number IAC 55272. The research project was registered in the Sistema de Patrimônio Genético e Conhecimento Tradicional Associado (Sisgen) platform, with the registration number A90708B.

### Extraction, Fractionation, and Purification of T-Cadinol

Dried and powdered leaves of *C. sylvestris* (290 g) were extracted using MeOH (5 × 1 L) at room temperature. After filtration and concentration under reduced pressure, the crude MeOH extract was resuspended in MeOH:H_2_O (9:1, v/v) and partitioned with *n*-hexane, CH_2_Cl_2_, EtOAc, and *n*-BuOH. These extracts were stored under refrigeration in an inert atmosphere and absence of light until its fractionation. After that, the *n*-hexane phase was evaluated and exhibited anti–*T. cruzi* activity (100% of trypomastigotes death at 300 μg/ml), so it was selected for fractionation procedures.

The hexane phase (6.4 g) was submitted by CC using SiO_2_ eluted with *n*-hexane with increased amounts of EtOAc to afford 10 fractions (I to X). Part of fraction V (1,076 mg) was subjected to CC over Sephadex LH-20 eluted with MeOH, to give four fractions (V-1 to V-4). Fraction V-2 (204.7 mg) was submitted to CC over SiO_2_ eluted with increased amounts of acetone in CH_2_Cl_2_ to give pure compound **1** (35 mg).

### Parasites and Mammalian Cell Maintenance

*T. cruzi* trypomastigotes (Y strain) were cultivated in the Rhesus monkey kidney cells (LLC-MK2, ATCC CCL 7) and were maintained in the RPMI-1640 medium (Sigma-Aldrich) supplemented with 2% FBS at 37°C in a 5% CO_2_ humidified incubator. Murine fibroblasts cells (NCTC clone L929, ATCC) and LLC-MK2 were maintained in the RPMI-1640 supplemented with 10% FBS at the same conditions described above. Macrophages were collected from the peritoneal cavity of BALB/c mice with RPMI-1640 supplemented with 10% FBS and were maintained in a 5% CO_2_ humidified incubator at 37°C.

### Evaluation of the Antitrypanosomal Activity

The solvent DMSO was used to dissolve the compounds. To avoid toxic effects in the parasites and mammalian cells, it was added at 0.5% (v/v) in the microplate wells and used as the internal control. The 50% inhibitory concentration (IC_50_) was determined against *T. cruzi* trypomastigote (*i*) and amastigote (*ii*) forms, as follows:

*i*) *Trypomastigotes*: Trypomastigotes (1 × 10^6^/well) were seeded in 96-well plates and incubated with compound **1** up to 150 μM for 24 h at 37°C in a 5% CO_2_ incubator. The parasite viability was determined using the resazurin (Alamar Blue^®^) colorimetric method. Benznidazole was used as the standard drug, and the untreated cells were used as the negative control (Barbosa et al., 2020). The mortality of the parasites induced by fractions (bioguided fractionation) was performed using the incubation of trypomastigotes at a fixed concentration of 300 µg/ml for 24 h at 37°C in a 5% CO_2_ incubator. The parasite viability was analyzed by light microscopy (× 400 magnification) by the motility and morphology of the parasites compared to the untreated group.
*ii*) *Amastigotes*: Peritoneal macrophages (1 × 10^5^ cells/well) were seeded in 16-well slide chambers (NUNC, Merck) and infected with trypomastigotes at a ratio of 10:1 (parasite/macrophage). After 2 h of incubation at 37°C in a 5% CO_2_ incubator, the cells were treated with compound **1** at different concentrations for 48 h. Benznidazole was used as the standard drug and the untreated cells as a negative control. Slides were stained with Giemsa, analyzed in a digital light microscope (EVOS M5000 Thermo Fisher, United States), and the IC_50_ values were calculated by the infection index (Londero et al., 2018).

### Cytotoxicity Against Mammalian Cells

NCTC cells (6 × 10^4^ cells/well), murine fibroblasts (ATCC), were seeded in 96-well plates and incubated with serial dilutions (base 2-fold) of compound **1** (200–1.6 μM) for 48 h in a 5% CO_2_ incubator at 37°C. The 50% cytotoxic concentration (CC_50_) was determined by the MTT colorimetric method (Tada et al., 1986). The selectivity index (SI) value was calculated using the ratio: CC_50_ against NCTC cells/IC_50_ against *T. cruzi* amastigotes.

### Evaluation of the Plasma Membrane Permeability

Trypomastigotes (2 × 10^6^ cells/well) were seeded in the 96-well black polystyrene microplates and incubated with 1 μM of SYTOX Green (Molecular Probes) in HANKS’ balanced salt solution (Sigma-Aldrich) supplemented with 10 mM d-Glucose (Sigma-Aldrich, HBSS + Glu) in the dark at 24°C. After 15 min, compound **1** was added at IC_50_ concentration (18.2 μM) and the fluorescence was monitored for 4 h using a fluorimetric microplate reader (FilterMax F5-Molecular Devices) with excitation and emission wavelengths of 485 and 535 nm, respectively. Maximum permeabilization was obtained with 0.5% Triton X-100 (positive control), and untreated parasites were considered as the negative control (Mesquita et al., 2014).

### Assessment of Plasma Membrane Electric Potential (ΔΨp)

Trypomastigotes (2 × 10^6^ cells/well) were treated with compound **1** (18.2 μM) in HBSS + Glu for 4 h at 37°C in a 5% CO_2_ incubator. Subsequently, 0.2 µM of DiSBAC_2_ 3) (Molecular Probes) were added and the samples were incubated at 37°C for 5 min. The fluorescence was measured using the Attune NxT flow cytometer (Thermo Fisher Scientific) with excitation and emission wavelengths of 488 and 574 nm, respectively. The parasites were treated with gramicidin D (0.5 µg/ml) (Sigma-Aldrich) (positive control) and the untreated parasites served as a negative control (Morais et al., 2019).

### Determination of Mitochondrial Membrane Electric Potential (ΔΨm)

Trypomastigotes (2 × 10^6^ cells/well) were incubated with compound **1** (18.2 μM) in HBSS + Glu for 4 h at 37°C in a 5% CO_2_ incubator. The parasites were stained with JC-1 dye (Thermo Fisher) at 10 μM in the dark for 20 min, and fluorescence levels were measured using the Attune NxT flow cytometer (Thermo Fisher Scientific) with an excitation filter wavelength of 488 nm, and emission wavelengths of 530 nm (BL-1) and 574 nm (BL-2). The ΔΨ_m_ was determined by the ratio BL-2/BL-1. CCCP (100 μM) (Sigma-Aldrich) treated parasites were used as a positive control and the untreated parasites as a negative control (Luque-Ortega and Rivas, 2010).

### Reactive Oxygen Species Analysis

Trypomastigotes (2 × 10^6^ cells/well) were treated with compound **1** (18.2 μM) for 4 h in HBSS + Glu at 37°C in a 5% CO_2_ incubator. H_2_DCFDA (molecular probe) was added (5 μM), and the parasites were incubated for 15 min. Then, the fluorescence intensity was measured using a fluorimetric microplate reader (FilterMax F5 Multi-Mode, Molecular Devices) with an excitation wavelength of 485 nm and an emission wavelength of 535 nm. Sodium azide (10 mM) was used as a positive control and the untreated parasites were used as a negative control (Keil et al., 2011).

### Assessment of Intracellular Calcium (Ca^2+^) Levels

Trypomastigotes (2 × 10^6^ cells/well) were pretreated with 5 μM of Fluo-4 AM (Molecular Probes) in PBS 1x, for 60 min at 37°C in the dark. Then, the parasites were washed and treated with compound **1** (18.2 μM). Fluorescence measurements were performed for 4 h using a fluorimetric microplate reader (FilterMax F5 Multi-Mode, Molecular Devices) with the excitation and emission wavelengths of 485 and 535 nm, respectively. Maximum calcium levels were obtained using 0.5% Triton X-100 and the untreated parasites were used as a negative control (Rea et al., 2013).

### Statistical Analysis

The 50% inhibitory concentrations and 50% cytotoxic concentration values were calculated using sigmoidal dose–response curves using GraphPad Prism 6.0 software. Unless stated, the reported data correspond to the mean ± standard deviation of at least two independent experiments performed with duplicate samples. The one-way ANOVA of variance with Tukey’s multiple comparison test was used for the significance test (*p* value).

## Results and Discussion

**FIGURE 1:**
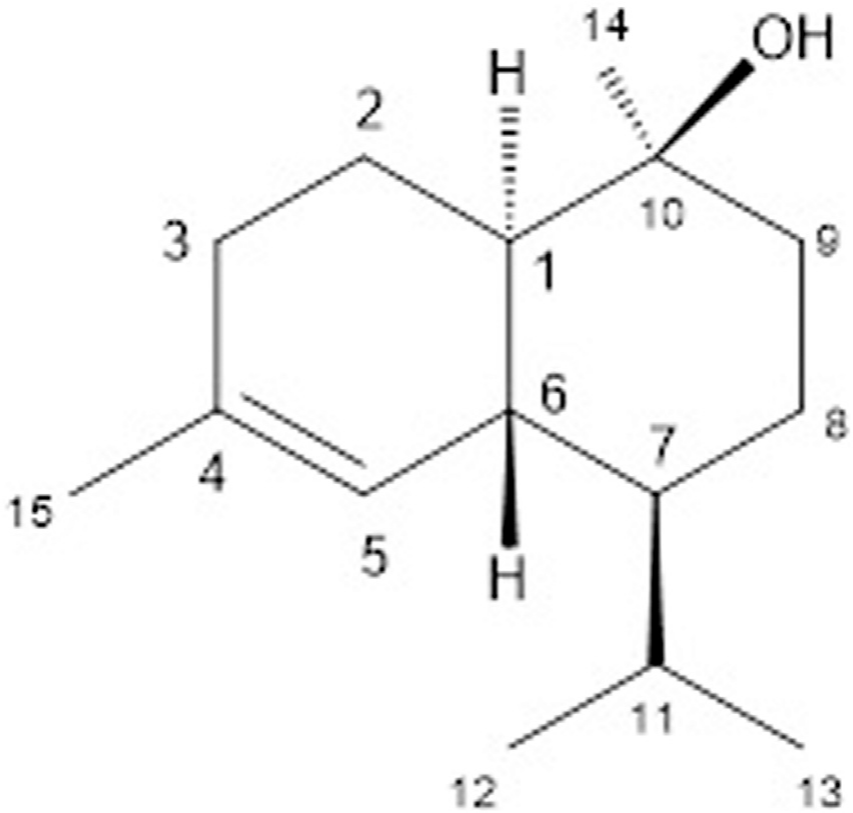
**-** Structure of (-)-Τ-cadinol (**1**).

To evaluate the anti–*T. cruzi* potential of the hexane phase from the MeOH extract of *C. sylvestris*, this material was tested *in vitro* against trypomastigote forms. By using bioguided fractionation to isolate only bioactive compounds, the parasites were incubated at 300 μg/ml, and the morphology and motility observed under light microscopy. This approach afforded a bioactive fraction composed by the sesquiterpene (-)-T-cadinol **1**) whose structure was identified based on the spectrometric analyses. The ESI–HRMS of compound **1** showed the [M + H]^+^ peak at *m/z* 223.2071, indicating a molecular formula C_15_H_26_O (calcd for C_15_H_27_O^+^ 223.2062).The ^1^H NMR spectrum of compound **1** displayed hydrogen signals belonging to the methyl groups as a pair of doublets at δ_H_ 0.74 and 0.87 (*J* = 6.9 Hz, 3H each), which were assigned to the methyl component of an isopropyl group (H-12 and H-13), besides, singlets at δ_H_ 1.09 (3H) and 1.60 (3H) are assigned to CH_3_-15 and CH_3_-14, respectively. Also, the spectrum showed a broad singlet at the δ_H_ 5.48 (1H) characteristic of an olefinic H-5, and four multiplets at δ_H_ 1.23, 1.96, 1.30, and 1.80 assigned to methynic hydrogen H-1, H-6, H-7, and H-11 respectively. Additional peaks, ranging from δ_H_ 1.54 to δ 1.96, correspond to the remaining hydrogen H-2, H-3, H-8, and H-9.The ^13^C and DEPT NMR spectra showed 15 carbon signals, corresponding to four methyl, four methylene, four methine, and three quaternary carbons, in order to confirming the occurrence of a sesquiterpene derivative. The cadinane skeleton was proposed due to the presence of signals attributed to sp^2^ carbons at δ_C_ 133.8 (C-4) and 123.4 (C-5), to the carbinolic carbon at δ_C_ 69.0 (C-10), and the methyl groups at δ_C_ 21.1 (C-12), 15.7 (C-13), 24.1 (C-14), and 29.0 (C-15). The comparison of the obtained data with those reported in the literature of (+)-T-cadinol (Labbe et al., 1993) allowed the identification of compound **1** as the enantiomer (-)-T-cadinol (Figure 1), since the isolated compound exhibited the opposite value of specific optical rotation ( [α] $\;\begin{array}{c} 20\\ D \end{array}$ -28; *c* 0,42 in CHCl_3_) of that reported to the (+)-isomer.

*Casearia* genus is known for the production of diterpenoids, especially those with the rearranged clerodane skeleton, known as casearins. Sesquiterpenes occur in a minority, and were well described in the essential oils from the leaves of *C. sylvestris* with biological activities against tumor cell lines (Bou et al., 2013; Pereira et al., 2017a), antileishmanial (Moreira et al., 2019), antifungal (Pereira et al., 2017b), gastric anti-ulcer, and anti-inflammatory (Esteves et al., 2005). Moreover, only two main compounds found in the essential oil from *C. sylvestris* were explored about their biological activity. BOU *et al.* (2013) isolated *α*-zingiberene from the essential oil of *C. sylvestris,* and this compound as well as some semi-synthetic derivatives showed cytotoxic potential against tumor cell lines. In addition, MOREIRA *et al.* (2019) reported the identification of *E*-caryophyllene in the essential oil of *C. sylvestris,* and both showed activity and selectivity to *Leishmania amazonensis* amastigotes (Moreira et al., 2019). The essential oil of *C. lasyophilla* leaves afforded *α*-cadinol (Salvador et al., 2011), an isomer of T-cadinol, isolated in the present work, from the bioactive *n*-hexane phase from the MeOH extract from the leaves of *C. sylvestris*. The antiparasitic activity of T-cadinol is discussed below, and corroborates the biological potential for the plant that is a rich source of bioactive prototypes, especially terpenoids (sequi and diterpenoids).

### Antitrypanosomal Activity

**FIGURE 2:**
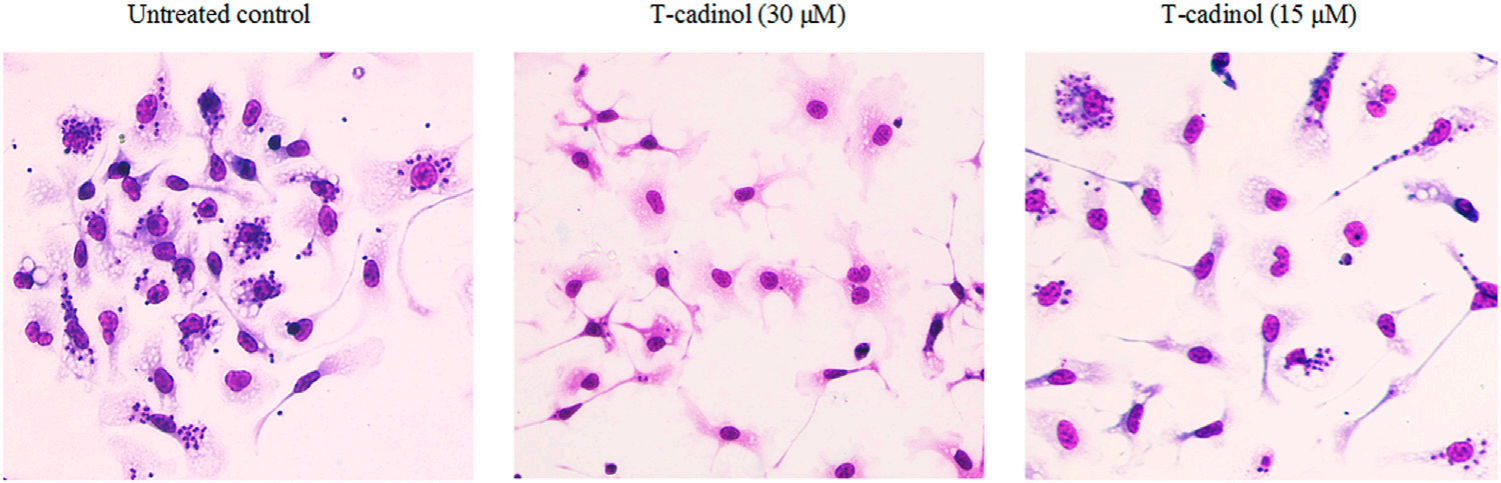
Light microscopy micrographs (× 400 magnification) of *T. cruzi*–infected macrophages treated with compound **1** at 30 and 15 µM. The untreated macrophages (*T.cruzi*–infected) were used as control. The images were acquired using the digital microscope EVOS M500 (Thermo-United States).

Compound **1** was evaluated *in vitro* against *T. cruzi.* and displayed activity against amastigotes and trypomastigotes forms of parasite with IC_50_ values of 15.8 and 18.2 µM, respectively (Table 1). Light microscopy analysis (×400 magnification) revealed that compound **1** eliminated the intracellular amastigotes with selectivity, killing >95% of the parasites at 30 µM (Figure 2), preserving the morphology of the host cells. At 15 μM, it was possible to observe 50% reduction of the amastigotes, when compared to the untreated macrophages (*T.cruzi*-infected) (Figure 2). The intracellular amastigotes are the clinically relevant forms of the parasite, and are mainly present in the chronic phase of the disease (Alonso-Padilla and Rodríguez, 2014); while the trypomastigotes is also of therapeutic importance, once this form is widely found at the acute phase of the human disease. If not completely eliminated during the drug treatment, trypomastigotes can lead to the reactivation of the parasitemia and disease transmission (Katsuno et al., 2015).

**TABLE 1:** **-** Anti–*T. cruzi* activity and mammalian cytotoxicity of compound **1** (T-cadinol).

Compound	IC_50_ (µM ± SD)		CC_50_ (µM ± SD)	SI
	Trypomastigote	Amastigote		
1	18.2 ± 7.7	15.8 ± 10.3	>200	>12
Benznidazole	17.7 ± 1.9	5.0 ± 1.5	190.6 ± 13.4	38.1

IC_50_, 50% inhibitory concentration; CC_50_, 50% cytotoxic concentration; SI, selectivity index (CC_50_ mammalian cells/IC_50_ amastigotes); SD, standard deviation.

Regarding the mammalian cytotoxicity, no toxic profile of the cells was detected after treatment with compound **1** to the highest tested concentration. Using the ratio between the cytotoxicity and activity against amastigotes, the selectivity index (SI) of this sesquiterpene was >12. Benznidazole showed an IC_50_ value against the intracellular amastigotes of 5.0 µM, and a mammalian cytotoxicity (CC_50_) of 190.6 µM, resulting in a selective index of 38.1*.*

Essential oils containing the T-cadinol (**1**) from different plants such as *Cymbopogon nardus* (Norhayati et al., 2013), *Kadsura longipedunculata* (Mulyaningsih et al., 2010), and *Humulus lupulus* (Jeliazkova et al., 2018) have demonstrated the activity against the etiologic agent of the African trypanosomiasis (*Trypanosoma brucei*). Considering the promising activity of T-cadinol against both forms of *T. cruzi* and the lack of mammalian cytotoxicity, we suggest that this compound could be used as a hit candidate for future optimization studies on drug discovery against Chagas disease.

### Mechanism of Action Studies

Considering the potent and selective activity of T-cadinol (**1**) against *T. cruzi*, we investigated the lethal action using the extracellular trypomastigotes. During drug discovery studies, the study of the target organelles of a hit candidate can provide significant information for the synthesis of new derivatives (Tiwari et al., 2018). The following assays were carried out in trypomastigotes at the 50% inhibitory concentration.

### Plasma Membrane Permeability

**FIGURE 3:**
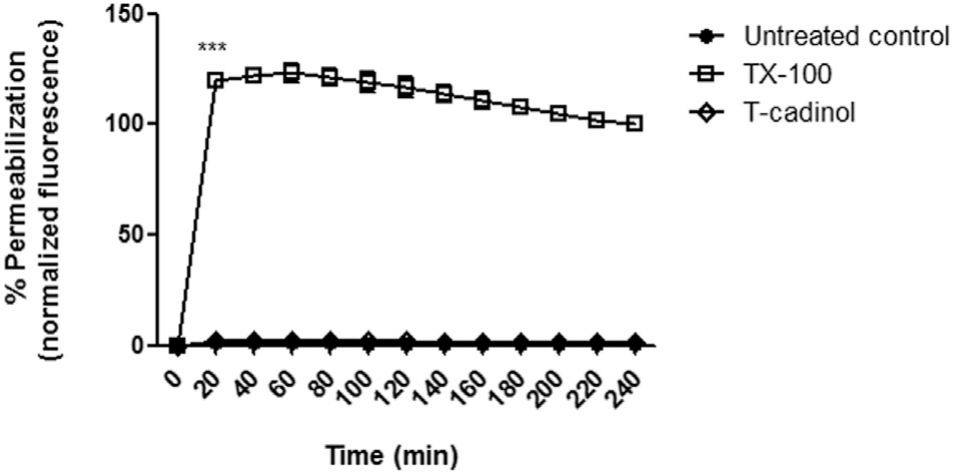
**–** Evaluation of plasma membrane permeabilization in *T. cruzi* trypomastigotes treated with compound 1 (*T-cadinol* −18.2 μM) for 4 h. Sytox^®^ Green dye fluorescence was monitored spectrofluorimetrically (excitation 485 nm and emission 535 nm) every 20 min. Untreated trypomastigotes and those treated with 0.5% TX-100 were used as negative and positive controls, respectively. Fluorescence is reported as the percentage relative to time 0 min (0%) and TX-100 240 min (100%). ***p < 0.0001.

The plasma membrane is an essential regulator of ions and nutrient transport, as well as pH homeostasis, acting as a barrier between the extra and intracellular environments. In the present study, the plasma membrane permeability of *T. cruzi* parasites was analyzed spectrofluorimetrically, using the probe SYTOX Green. Our results showed that compound **1** induced no permeabilization of the membrane when compared to the untreated parasites (Figure 3), similar to the eudesmane sesquiterpene costic acid, isolated from *Nectandra barbellata* (Lauraceae) (Londero et al., 2020).

### Plasma Membrane Electric Potential (ΔΨp)

**FIGURE 4:**
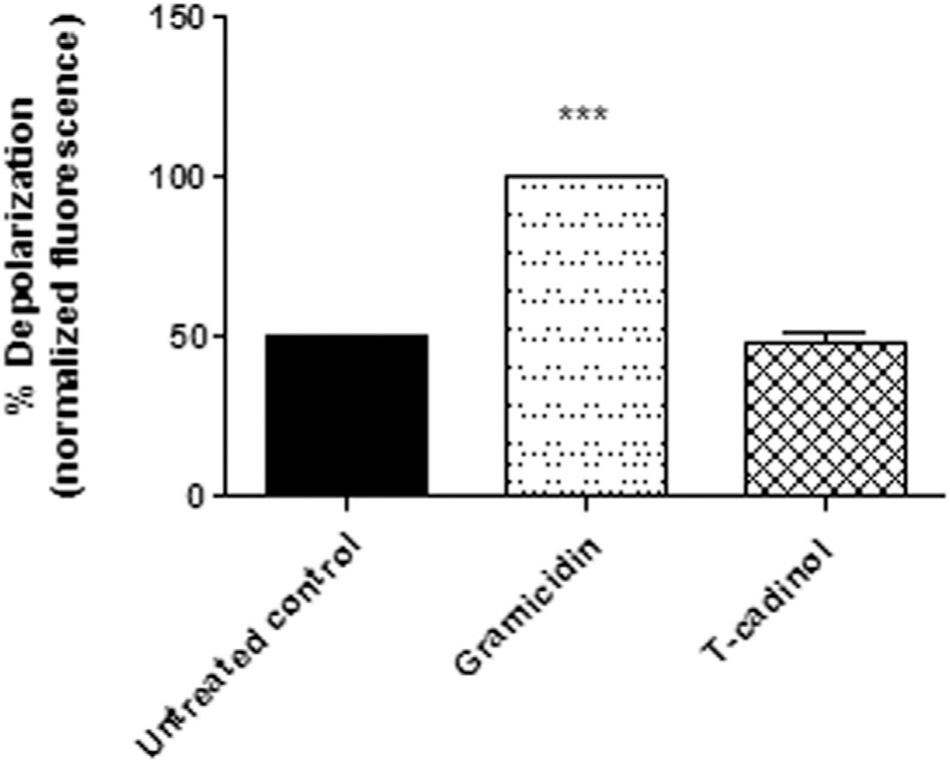
**–** Evaluation of plasma membrane electric potential (ΔΨp) in *T. cruzi* trypomastigotes treated with compound **1** (T-cadinol −18.2 μM) for 4 h. DISBAC2(3) dye fluorescence was measured by flow cytometry (excitation 488 nm and emission 574 nm). Untreated trypomastigotes were used to achieve minimal depolarization and treated with gramicidin D (0.5 µg/ml) to achieve the maximum. Fluorescence was normalized with respect to gramicidin D (100%). ***p < 0.0001.

The membrane potential and the differences in ion concentrations between the intracellular and the extracellular millie determine the flow of ions in channels. Alterations in the potential can lead to an ionic imbalance and the formation of transmembrane pores, affecting the acquisition of crucial nutrients for proliferation and cellular activities (Morth et al., 2011). In the present work, using the fluorophore DiSBAC_2_ in flow cytometry, it was possible to verify that the compound **1** caused no interference in the membrane when compared to untreated parasites (Figure 4). It suggests that T-cadinol enters the plasma membrane of the parasite without affecting the gradient of cations (Na^+^, Ca^2+^, and K^+^) and anions Cl^−^,that forces ions to passively move in one direction. Considering the lethal activity of T-cadinol (**1**) in the trypomastigotes, our initial approaches suggest other mechanisms of action of the compound than the parasite plasma membrane damage.

Protozoan parasites present membrane structures with considerable differences in lipids and proteins relative to the mammalian cells. These are essential for parasite specific processes, such as host cell invasion, nutrient acquisition or protection against the host immune system (Vial et al., 2003). Despite the attractive approach to target *T. cruzi* membrane with natural products, compound **1** showed no interference in this site and we proceeded to investigate other organelles.

### Mitochondrial Membrane Electric Potential (ΔΨm)

From energy production to oxidative stress control, the mitochondria is responsible for innumerable metabolic processes. This organelle is involved in the growth and differentiation, being crucial to the cell survival. Additionally, unlike mammalian cells, trypanosomatids exhibit a unique mitochondria, which extends throughout the parasite length (Menna–Barreto and Medeiros, 2014). Due to these factors, the search for compounds that affect this essential organelle could be an interesting strategy for the search of new anti–*T. cruzi* therapies.

**FIGURE 5:**
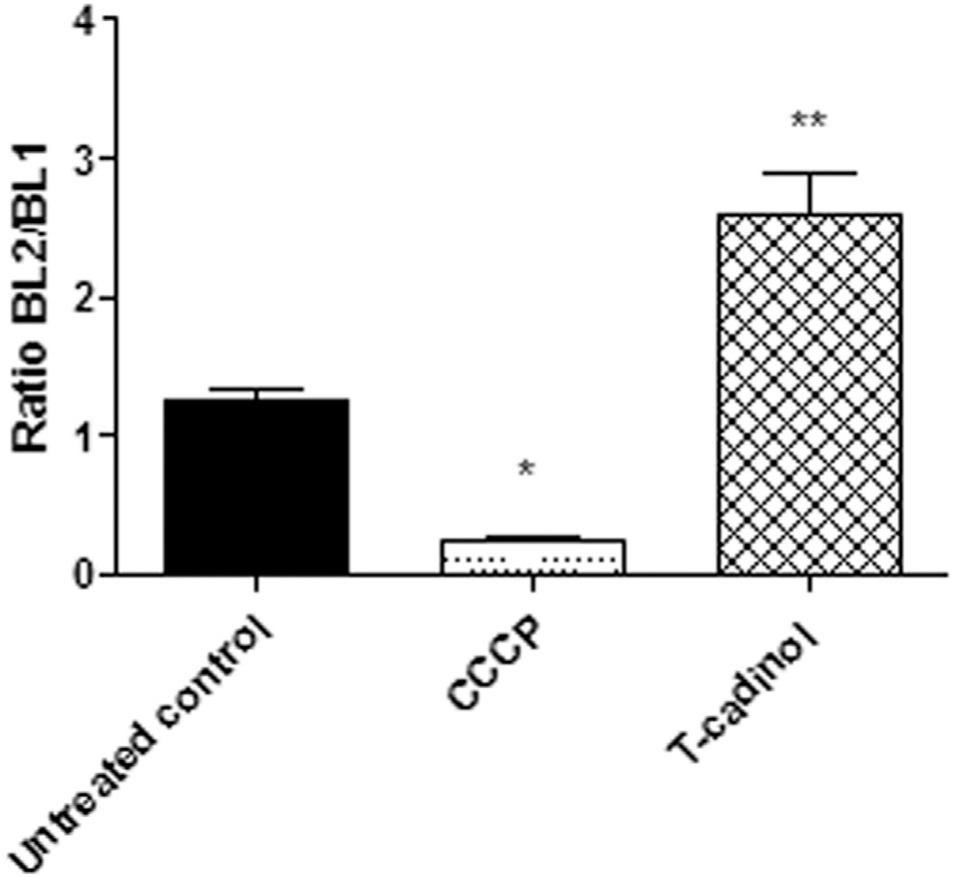
**-** Evaluation of mitochondrial membrane potential (∆ψ_m_) in *T. cruzi* trypomastigotes treated with compound **1** (T-cadinol −18.2 μM) for 4 h. JC-1 dye fluorescence was monitored by flow cytometry (excitation 488 nm and emission 530/574 nm). Untreated trypomastigotes and those treated with CCCP (100 μM) were used to achieve minimal and maximal depolarization, respectively. Fluorescence is reported as the ratio between the emission channels BL2/BL1. **p < 0.0021 and *p*<*0.05.

To analyze the mitochondrial membrane of *T. cruzi* trypomastigotes, the fluorescence of JC-1 dye was used in the flow cytometer. This fluorophore indicates changes in the mitochondrial membrane potential according to the formation of J-aggregates (BL-2 fluorescence) and monomers (BL-1 fluorescence). Our data showed an intense hyperpolarization of the mitochondrial membrane, leading to a significant enhance in the BL-2/BL-1 ratio when compared to the untreated parasites (Figure 5). Previous reports of two other natural sesquiterpenes, costic acid isolated from *Nectandra barbellata* (Londero et al., 2020) and deoxymikanolide isolated from the species of *Mikania* (Puente et al., 2019), demonstrated an imbalance of the *T. cruzi* mitochondria, but leading to a depolarization of this organelle. The mitochondria in trypanosomatid are unique and vital organelles, serving as target for several drugs and compounds. The current findings established the essential role of the mitochondria in protozoan parasites and their peculiarities as compared to the mammalian counterpart, which is being considered an attractive candidate for drug discovery (Sundar and Singh, 2018).

### Reactive Oxygen Species

**FIGURE 6:**
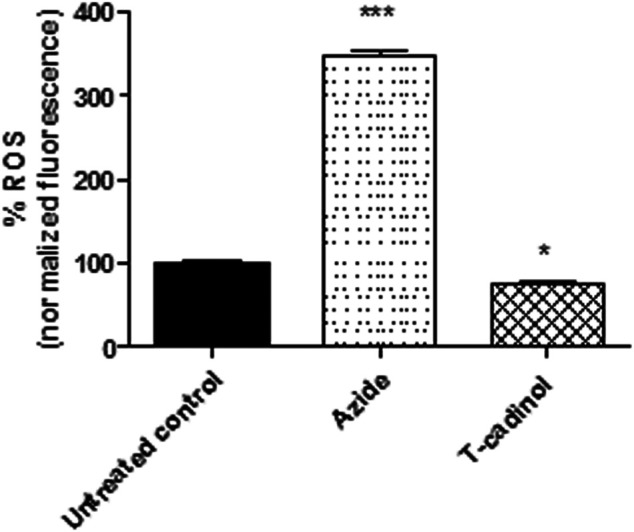
**-** Evaluation of reactive oxygen species (ROS) production in *T. cruzi* trypomastigotes treated with compound **1** (T-cadinol −18.2 μM) for 4 h H_2_DCFDA dye fluorescence was measured spectrofluorimetrically (excitation 485 nm and emission 535 nm). Untreated trypomastigotes were used as a negative control, and those treated with sodium azide (10 mM) as a positive control. Fluorescence was normalized with respect to untreated trypomastigotes (100%). ***p < 0.0001 and *p*<*0.05.

Mitochondria are the principal resource of reactive oxygen species such as superoxide anions, hydrogen peroxide, and hydroxyl radicals. Under normal conditions, the respiratory chain generates around 3–5% of ROS over the total oxygen consumed, and these species are used as signalization in the cell proliferation and growth. Therefore, derangements in the mitochondria can affect the production of these species, caused from interference in different biosynthetic pathways to lethal oxidative stress (Machado-Silva et al., 2016; Maldonado et al., 2020). Considering the effect of compound **1** in the mitochondria, the ROS levels in *T. cruzi* using the fluorescent probe H_2_DCFDA was also investigated. The results demonstrated that compound **1** induced a slight decrease in the ROS levels, characterizing a reduced fluorescence when compared to the untreated parasites (Figure 6). This reduction can be attributed to the mitochondrial membrane hyperpolarization, since the breakdown of the respiratory chain can cause a disorder of the proton motive force and consequently, the alteration of the ROS production.

The function of the mitochondria is the production of metabolic energy in the form of ATP. Most of the oxygen consumed by the mitochondrial electron transport chain is reduced to water, and a small proportion is converted to ROS (1–2%) (Wiley et al., 2014). The reduction of ROS caused by the compound 1 suggests a damage to the bioenergetic activity of *T. cruzi* mitochondria, as corroborated by the studies of the *ΔΨ*_*m.*_

### Intracellular Calcium (Ca^2+^)

Intracellular calcium is an important cell signaling component and plays a key role in trypanosomatids infectivity, mobility, and differentiation. These ions are concentrated mostly in the endoplasmic reticulum, mitochondria, and acidocalcisomes. Basically, failure in any Ca^2+^ regulation pathway or storage disarranges homeostasis and can be extremely harmful to the parasites. Additionally, variations in the mitochondrial calcium induce high conductance channel formation across the membrane, disturbing the electrical potential (Benaim et al., 2020a; Scarpelli et al., 2021).

**FIGURE 7:**
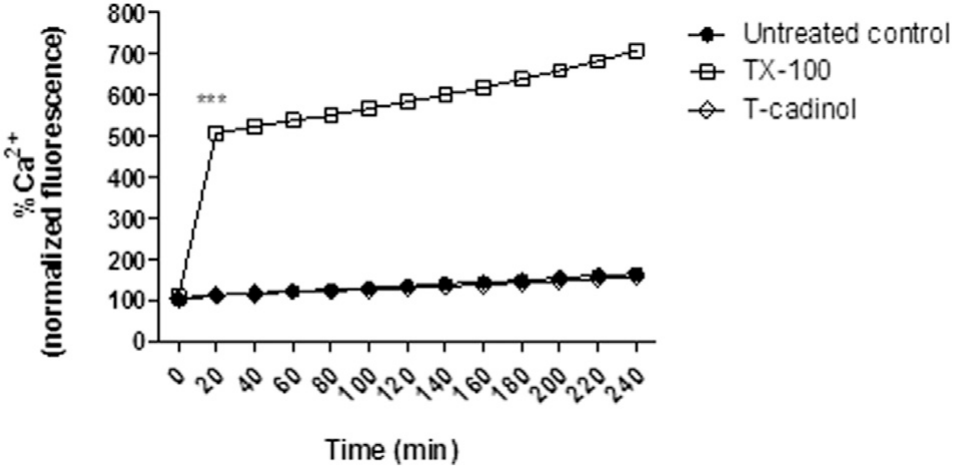
**-** Evaluation of intracellular Ca^2+^ levels in *T. cruzi* trypomastigotes treated with compound **1** (T-cadinol −18.2 μM) for 4 h. Fluo-4 AM dye fluorescence was monitored spectrofluorimetrically (excitation 485 nm and emission 535 nm). Untreated trypomastigotes and those treated with 0.5% TX-100 were used as negative and positive controls, respectively. Fluorescence is reported as the percentage relative to time 0 min (100%). ***p < 0.0001.

In the present work, Ca^2+^ levels were spectrofluorimetrically evaluated employing the Fluo-4 AM dye. The obtained results showed that compound **1** caused no interference in the intracellular calcium levels of *T. cruzi* when compared to the untreated parasites (Figure 7). Based on these findings, it is possible to assume that the mitochondrial impairment caused by *T-cadinol* (**1**) has no direct relationship to a Ca^2+^ extrusion effect. Further studies should be performed to clarify if T-cadinol (**1**) has a direct effect in the mitochondria or can also affect other metabolic pathways at early time of incubation. Despite Ca^2+^ imbalance has been considered an attractive strategy against *Trypanosoma cruzi* parasites, both the single mitochondria and the acidocalcisomes have been considered important targets for drug discovery studies, as they play essential roles in the bioenergetics of these protozoan parasites (Benaim et al., 2020b).

## Conclusion

In the present work, *C. sylvestris* was found to be an important source of anti–*T. cruzi* natural products with T-cadinol (**1**) isolated for the first time from this plant. We also demonstrated unprecedentedly the selective and potent activity of compound **1** against amastigote and trypomastigote forms of *T. cruzi*, with no cytotoxicity to mammalian cells. The mechanism of the action analysis of compound **1** showed that the mitochondria are affected, but future studies are needed to confirm this organelle as a candidate target. Overall, T-cadinol (**1**) is a promising scaffold for optimization studies in drug discovery to the treatment of Chagas disease.

## Data Availability

The original contributions presented in the study are included in the article/Supplementary Material; further inquiries can be directed to the corresponding authors.

## References

[B1] Alonso-Padilla J. Rodríguez A. (2014). High Throughput Screening for Anti-trypanosoma Cruzi Drug Discovery. Plos Negl. Trop. Dis. 8, e3259. 10.1371/journal.pntd.0003259 25474364 PMC4256210

[B2] Ameni A. Z. Latorre O. A. Torres L. M. Górniak S. L. (2015). Toxicity Study about a Medicinal Plant *Casearia Sylvestris*: A Contribution to the Brazilian Unified Health System (SUS). J. Ethnopharmacol 175, 9–13. 10.1016/j.jep.2015.08.027 26344853

[B3] Antinarelli L. M. Pinto N. C. Scio E. Coimbra E. S. (2015). Antileishmanial Activity of Some Brazilian Plants, with Particular Reference to *Casearia Sylvestris* . Acad. Bras Cienc 87, 733–742. 10.1590/0001-3765201520140288 26062121

[B4] Barbosa H. da Silva R. L. C. G. Costa-Silva T. A. Tempone A. G. Antar G. M. Lago J. H. G. (2020). Interaction of Dicentrinone, an Antitrypanosomal Aporphine Alkaloid Isolated from *Ocotea Puberula* (Lauraceae), in Cell Membrane Models at the Air-Water Interface. Bioorg. Chem. 101, 103978. 10.1016/j.bioorg.2020.103978 32534347

[B5] Benaim G. Paniz-Mondolfi A. E. Sordillo E. M. Martinez-Sotillo N. (2020a). Disruption of Intracellular Calcium Homeostasis as a Therapeutic Target against *Trypanosoma Cruzi* . Front Cel Infect Microbiol 10, 46–15. 10.3389/fcimb.2020.00046 PMC7040492 32133302

[B6] Benaim G. Paniz-Mondolfi A. E. Sordillo E. M. Martinez-Sotillo N. (2020b). Disruption of Intracellular Calcium Homeostasis as a Therapeutic Target against *Trypanosoma Cruzi* . Front. Cel Infect Microbiol 10, 46. 10.3389/fcimb.2020.00046 PMC7040492 32133302

[B7] Bou D. D. Lago J. H. Figueiredo C. R. Matsuo A. L. Guadagnin R. C. Soares M. G. (2013). Chemical Composition and Cytotoxicity Evaluation of Essential Oil from Leaves of Casearia Sylvestris, its Main Compound α-zingiberene and Derivatives. Molecules 18, 9477–9487. 10.3390/molecules18089477 23966073 PMC6269751

[B8] Bou D. D. Tempone A. G. Pinto É. G. Lago J. H. Sartorelli P. (2014). Antiparasitic Activity and Effect of Casearins Isolated from *Casearia Sylvestris* on *Leishmania* and *Trypanosoma Cruzi* Plasma Membrane. Phytomedicine 21, 676–681. 10.1016/j.phymed.2014.01.004 24560122

[B9] De Rycker M. Baragaña B. Duce S. L. Gilbert I. H. (2018). Challenges and Recent Progress in Drug Discovery for Tropical Diseases. Nature 559, 498–506. 10.1038/s41586-018-0327-4 30046073 PMC6129172

[B10] Esteves I. Souza I. R. Rodrigues M. Cardoso L. G. Santos L. S. Sertie J. A. (2005). Gastric Antiulcer and Anti-inflammatory Activities of the Essential Oil from *Casearia Sylvestris* Sw. J. Ethnopharmacol 101, 191–196. 10.1016/j.jep.2005.04.020 15994044

[B11] Ferreira P. M. Costa-Lotufo L. V. Moraes M. O. Barros F. W. Martins A. M. Cavalheiro A. J. (2011). Folk Uses and Pharmacological Properties of *Casearia Sylvestris*: a Medicinal Review. Acad. Bras Cienc 83, 1373–1384. 10.1590/s0001-37652011005000040 22159347

[B12] Ferreira P. M. Militão G. C. Lima D. J. Costa N. D. Machado Kda. C. Santos A. G. (2014). Morphological and Biochemical Alterations Activated by Antitumor Clerodane Diterpenes. Chem. Biol. Interact 222, 112–125. 10.1016/j.cbi.2014.10.015 25452174

[B13] Ferreira P. M. P. Bezerra D. P. Silva J. D. N. da Costa M. P. Ferreira J. R. O. Alencar N. M. N. (2016). Preclinical Anticancer Effectiveness of a Fraction from *Casearia Sylvestris* and its Component Casearin X: *In Vivo* and *Ex Vivo* Methods and Microscopy Examinations. J. Ethnopharmacol 186, 270–279. 10.1016/j.jep.2016.04.011 27067367

[B14] Grandi T. S. M. (2014). Tratado das plantas medicinais: mineiras, nativas e cultivadas. Adequatio Estúdio, 978–985. 58322-00-0.

[B15] Itokawa H. Totsuka N. Morita H. Takeya K. Iitaka Y. SchenkeL E. P. (1990). New Antitumor Principles, Casearins A-F, for *Casearia Sylvestris* Sw. (Flacourtiaceae). Chem. Pharm. Bull. (Tokyo) 38, 3384–3388. 10.1248/cpb.38.3384 2092935

[B16] Jeliazkova E. D Zheljazkov V. Kačániova M. Astatkie T. L Tekwani B. (2018). Sequential Elution of Essential Oil Constituents during Steam Distillation of Hops (*Humulus Lupulus* L.) and Influence on Oil Yield and Antimicrobial Activity. J. Oleo Sci. 67, 871–883. 10.5650/jos.ess17216 29877222

[B17] Katsuno K. Burrows J. N. Duncan K. Hooft van Huijsduijnen R. Kaneko T. Kita K. (2015). Hit and lead Criteria in Drug Discovery for Infectious Diseases of the Developing World. Nat. Rev. Drug Discov. 14, 751–758. 10.1038/nrd4683 26435527

[B18] Keil V. C. Funke F. Zeug A. Schild D. Müller M. (2011). Ratiometric High-Resolution Imaging of JC-1 Fluorescence Reveals the Subcellular Heterogeneity of Astrocytic Mitochondria. Pflugers Arch. 462, 693–708. 10.1007/s00424-011-1012-8 21881871 PMC3192276

[B19] Kratz J. M. (2019). Drug Discovery for Chagas Disease: A Viewpoint. Acta Trop. 198, 105107. 10.1016/j.actatropica.2019.105107 31351074

[B20] Labbe C. Castillo M. Connolly J. D. (1993). Mono and Sesquiterpenoids from *Satureja Gilliesii* . Phytochemistry 34, 441–444. 10.1016/0031-9422(93)80026-o

[B21] Londero V. S. Costa-Silva T. A. Tempone A. G. Namiyama G. M. Thevenard F. Antar G. M. (2020). Anti-*Trypanosoma Cruzi* Activity of Costic Acid Isolated from *Nectandra Barbellata* (Lauraceae) Is Associated with Alterations in Plasma Membrane Electric and Mitochondrial Membrane Potentials. Bioorg. Chem. 95, 103510. 10.1016/j.bioorg.2019.103510 31884137

[B22] Londero V. S. da Costa-Silva T. A. Gomes K. S. Ferreira D. D. Mesquita J. T. Tempone A. G. (2018). Acetylenic Fatty Acids from *Porcelia Macrocarpa* (Annonaceae) against Trypomastigotes of *Trypanosoma Cruzi*: Effect of Octadec-9-Ynoic Acid in Plasma Membrane Electric Potential. Bioorg. Chem. 78, 307–311. 10.1016/j.bioorg.2018.03.025 29625270

[B23] Luque-Ortega J. R. Rivas L. (2010). Characterization of the Leishmanicidal Activity of Antimicrobial Peptides. Methods Mol. Biol. 618, 393–420. 10.1007/978-1-60761-594-1_25 20094878

[B24] Machado-Silva A. Cerqueira P. G. Grazielle-Silva V. Gadelha F. R. Peloso Ede. F. Teixeira S. M. (2016). How *Trypanosoma Cruzi* Deals with Oxidative Stress: Antioxidant Defence and DNA Repair Pathways. Mutat. Res. Rev. Mutat. Res. 767, 8–22. 10.1016/j.mrrev.2015.12.003 27036062

[B25] Maldonado E. Rojas D. A. Morales S. Miralles V. Solari A. (2020). Dual and Opposite Roles of Reactive Oxygen Species (ROS) in Chagas Disease: Beneficial on the Pathogen and Harmful on the Host. Oxid Med. Cel Longev 2020, 8867701. 10.1155/2020/8867701 PMC7746463 33376582

[B26] Marquete R. Medeiros E. V. S. S. (2020). Salicaceae in Flora Do Brasil 2020 Em Construção. Available at: http://floradobrasil.jbrj.gov.br/reflora/floradobrasil/FB14361>. Acesso em (Disponível em September 14, 2021).

[B27] Menna-Barreto R. F. de Castro S. L. (2014). The Double-Edged Sword in Pathogenic Trypanosomatids: the Pivotal Role of Mitochondria in Oxidative Stress and Bioenergetics. Biomed. Res. Int. 2014, 614014–14. 10.1155/2014/614014 24800243 PMC3988864

[B28] Mesquita J. T. da Costa-Silva T. A. Borborema S. E. Tempone A. G. (2014). Activity of Imidazole Compounds on *Leishmania* (L.) *Infantum Chagasi*: Reactive Oxygen Species Induced by Econazole. Mol. Cel Biochem 389, 293–300. 10.1007/s11010-013-1954-6 24374794

[B29] Mesquita M. L. Desrivot J. Bories C. Fournet A. Paula J. E. Grellier P. (2005). Antileishmanial and Trypanocidal Activity of Brazilian Cerrado Plants. Mem. Inst. Oswaldo Cruz 100, 783–787. 10.1590/s0074-02762005000700019 16419337

[B30] Morais T. R. Costa-Silva T. A. Ferreira D. D. Novais B. J. Torrecilhas A. C. T. Tempone A. G. (2019). Antitrypanosomal Activity and Effect in Plasma Membrane Permeability of (-)-bornyl P-Coumarate Isolated from Piper Cernuum (Piperaceae). Bioorg. Chem. 89, 103001. 10.1016/j.bioorg.2019.103001 31129501

[B31] Moreira R. R. D. Santos A. G. d. Carvalho F. A. Perego C. H. Crevelin E. J. Crotti A. E. M. (2019). Antileishmanial Activity of Melampodium Divaricatum and Casearia Sylvestris Essential Oils on Leishmania Amazonensis. Revista do Instituto de Medicina Trop. de São Paulo 61, e33. 10.1590/s1678-9946201961033 PMC6609133 31269109

[B32] Morth J. P. Pedersen B. P. Buch-Pedersen M. J. Andersen J. P. Vilsen B. Palmgren M. G. (2011). A Structural Overview of the Plasma Membrane Na+,K+-ATPase and H+-ATPase Ion Pumps. Nat. Rev. Mol. Cel Biol 12, 60–70. 10.1038/nrm3031 21179061

[B33] Mulyaningsih S. Youns M. El-Readi M. Z. Ashour M. L. Nibret E. Sporer F. (2010). Biological Activity of the Essential Oil of *Kadsura Longipedunculata* (Schisandraceae) and its Major Components. J. Pharm. Pharmacol. 62, 1037–1044. 10.1111/j.2042-7158.2010.01119.x 20663038

[B34] Newman D. J. Cragg G. M. (2020). Natural Products as Sources of New Drugs over the Nearly Four Decades from 01/1981 to 09/2019. J. Nat. Prod. 83, 770–803. 10.1021/acs.jnatprod.9b01285 32162523

[B35] Muhd Haffiz J. Norhayati I. Getha K. Nor Azah M. A. Mohd Ilham A. Lili Sahira H. (2013). Chemical Composition and *In Vitro* Antitrypanosomal Activity of Fractions of Essential Oil from *Cymbopogon Nardus* L. Trop. Biomed. 30 (1), 9–14. 23665703

[B36] Pereira F. G. Marquete R. Domingos L. T. Rocha M. E. N. Ferreira-Pereira A. Mansur E. (2017a). Antifungal Activities of the Essential Oil and its Fractions Rich in Sesquiterpenes from Leaves of *Casearia Sylvestris* Sw. Acad. Bras Cienc 89, 2817–2824. 10.1590/0001-3765201720170339 29236852

[B37] Pereira F. G. Marquete R. Oliveira-Cruz L. Quintanilha-Falcao D. Mansur E. Moreira D. D. (2017b). Cytotoxic Effects of the Essential Oil from Leaves of *Casearia Sylvestris* Sw. (Salicaceae) and its Nanoemulsion on A549 Tumor Cell Line. Bol Latinoam. Caribe Plantas Med. Aromát. 16, 506–512.

[B38] Puente V. Laurella L. C. Spina R. M. Lozano E. Martino V. S. Sosa M. A. (2019). Primary Targets of the Sesquiterpene Lactone Deoxymikanolide on *Trypanosoma Cruzi* . Phytomedicine 56, 27–34. 10.1016/j.phymed.2018.10.015 30668348

[B39] Rea A. Tempone A. G. Pinto E. G. Mesquita J. T. Rodrigues E. Silva L. G. (2013). Soulamarin Isolated from Calophyllum Brasiliense (Clusiaceae) Induces Plasma Membrane Permeabilization of Trypanosoma Cruzi and Mytochondrial Dysfunction. Plos Negl. Trop. Dis. 7, e2556. 10.1371/journal.pntd.0002556 24340110 PMC3854968

[B40] Ribeiro V. Dias N. Paiva T. Hagström-Bex L. Nitz N. Pratesi R. (2020). Current Trends in the Pharmacological Management of Chagas Disease. Int. J. Parasitol. Drugs Drug Resist. 12, 7–17. 10.1016/j.ijpddr.2019.11.004 31862616 PMC6928327

[B41] Salvador M. J. Carvalho J. E. d. Wisniewski-Jrm A. Kassuya C. A. L. Santos É. P. Riva D. (2011). Chemical Composition and Cytotoxic Activity of the Essential Oil from the Leaves of *Casearia Lasiophylla* . Rev. Bras. Farmacogn. 21, 864–868. 10.1590/s0102-695x2011005000073

[B42] Scarpelli P. H. Pecenin M. F. Garcia C. R. S. (2021). Intracellular Ca2+ Signaling in Protozoan Parasites: An Overview with a Focus on Mitochondria. Ijms 22, 469. 10.3390/ijms22010469 PMC7796463 33466510

[B43] Sundar S. Singh B. (2018). Emerging Therapeutic Targets for Treatment of Leishmaniasis. Expert Opin. Ther. Targets 22, 467–486. 10.1080/14728222.2018.1472241 29718739 PMC6047532

[B44] Tada H. Shiho O. Kuroshima K. Koyama M. Tsukamoto K. (1986). An Improved Colorimetric Assay for Interleukin 2. J. Immunol. Methods 93, 157–165. 10.1016/0022-1759(86)90183-3 3490518

[B45] Tiwari N. Gedda M. R. Tiwari V. K. Singh S. P. Singh R. K. (2018). Limitations of Current Therapeutic Options, Possible Drug Targets and Scope of Natural Products in Control of Leishmaniasis. Mini Rev. Med. Chem. 18, 26–41. 10.2174/1389557517666170425105129 28443518

[B46] Vial H. J. Eldin P. Tielens A. G. van Hellemond J. J. (2003). Phospholipids in Parasitic Protozoa. Mol. Biochem. Parasitol. 126, 143–154. 10.1016/s0166-6851(02)00281-5 12615313

[B47] WHO (2017). Integrating Neglected Tropical Diseases into Global Health and Development: Fourth WHO Report on Neglected Tropical Diseases. Geneva: World Health Organization.

[B48] Wiley L. Ashok D. Martin-Ruiz C. Talbot D. C. Collerton J. Kingston A. (2014). Reactive Oxygen Species Production and Mitochondrial Dysfunction in White Blood Cells Are Not Valid Biomarkers of Ageing in the Very Old. PLOS ONE 9, e91005. 10.1371/journal.pone.0091005 24614678 PMC3948743

[B49] Xia L. Guo Q. Tu P. Chai X. (2015). The Genus *Casearia*: a Phytochemical and Pharmacological Overview. Phytochem. Rev. 14, 99–135. 10.1007/s11101-014-9336-6

