# Corrigendum: (-)-T-Cadinol, a Sesquiterpene Isolated From *Casearia sylvestris* (Salicaceae)—Displayed *In Vitro* Activity and Causes Hyperpolarization of the Membrane Potential of *Trypanosoma cruzi*

**DOI:** 10.3389/fphar.2022.865432

**Published:** 2022-03-14

**Authors:** Augusto L. dos Santos, Maiara Amaral, Flavia Rie Hasegawa, João Henrique G. Lago, Andre G. Tempone, Patricia Sartorelli

**Affiliations:** ^1^ Instituto de Ciências Ambientais, Químicas e Farmacêuticas, Universidade Federal de São Paulo, Diadema, Brazil; ^2^ Centro de Parasitologia e Micologia, Instituto Adolfo Lutz, Santo André, Brazil; ^3^ Faculdade de Medicina, Universidade de São Paulo, São Paulo, Brazil; ^4^ Centro de Ciências Naturais e Humanas, Universidade Federal do ABC, Santo André, Brazil

**Keywords:** *Trypanosoma cruzi*, *Casearia sylvestris*, Salicaceae, T-cadinol, Chagas disease

In the original article, there is an error in the Acknowledgments statement. The correct numbers for FAPESP are 2021/02789-7, 2016/24985-4 and 2018/10279-6. The corrected Acknowledgments statement appears below:

